# The Relations of Pelvic Disease to Physical Disturbances in Women

**Published:** 1892-12

**Authors:** 


THE RELATIONS OF PELVIC DISEASE TO PHYSICAL DISTURBANCES IN WOMEN.—Dr. George H. Rohe, of Catonsville, Md., read a paper on this subject at the fifth annual meeting of the American Association of Obstetricians and Gynecologists, at St. Louis, Mo., September 20–23, 1892, pointing out the frequency with which bodily conditions influenced mental states.

In a hospital containing 200 insane women, twenty-six were found with evidence of pelvic diseases. In eighteen of these, the uterine appendages were removed with the following results:

Sixteen recovered from the operation and two died. Of the sixteen recovered, three have been discharged from the hospital completely restored, both physically and mentally. In ten, considerable improvement followed the operation in both physical and mental conditions, and in three the operation was of too recent a date to allow any definite expression of opinion.

The mental disorder present in the eighteen cases was melancholia in six cases, simple mania in one, puerperal mania in four, hysterical mania in one, periodic mania in two, hystero-epilepsy with mania in one, and epilepsy with mania in three.

The author basing his opinion upon his experience, concludes as follows :

"The facts recorded demonstrate, first, that there is a fruitful field for gynecological work among insane women; second, that this work is as practicable and can be pursued with as much success in an insane hospital as elsewhere; and third, that the results obtained not only encourage us to continue in the work, but require us, in the name of science and humanity to give to an insane woman the same chance of relief from disease of the ovaries and uterus that a sane woman has.—*Alienist and Neurologist.*

